# Differential Phenotypes of Myeloid-Derived Suppressor and T Regulatory Cells and Cytokine Levels in Amnestic Mild Cognitive Impairment Subjects Compared to Mild Alzheimer Diseased Patients

**DOI:** 10.3389/fimmu.2017.00783

**Published:** 2017-07-07

**Authors:** Aurélie Le Page, Hugo Garneau, Gilles Dupuis, Eric H. Frost, Anis Larbi, Jacek M. Witkowski, Graham Pawelec, Tamàs Fülöp

**Affiliations:** ^1^Faculty of Medicine and Health Sciences, Research Center on Aging, Graduate Program in Immunology, University of Sherbrooke, Sherbrooke, QC, Canada; ^2^Faculty of Medicine and Health Sciences, Department of Biochemistry, Graduate Program in Immunology, University of Sherbrooke, Sherbrooke, QC, Canada; ^3^Faculty of Medicine and Health Sciences, Department of Infectious Diseases and Microbiology, Graduate Program in Immunology, University of Sherbrooke, Sherbrooke, QC, Canada; ^4^A*STAR, Singapore Immunology Network, Singapore, Singapore; ^5^Department of Pathophysiology, Medical University of Gdańsk, Gdańsk, Poland; ^6^Department of Internal Medicine II, Center for Medical Research University of Tübingen, Tübingen, Germany; ^7^Health Sciences North Research Institute, Sudbury, ON, Canada

**Keywords:** Alzheimer’s disease, myeloid-derived suppressor cells, regulatory T cell, amnestic mild cognitive impairment patients, cytokines, inflammation

## Abstract

Alzheimer disease (AD) is the most prevalent form of dementia although the underlying cause(s) remains unknown at this time. However, neuroinflammation is believed to play an important role and suspected contributing immune parameters can be revealed in studies comparing patients at the stage of amnestic mild cognitive impairment (aMCI) to healthy age-matched individuals. A network of immune regulatory cells including regulatory T cells (Tregs) and myeloid-derived suppressor cells (MDSCs) maintains immune homeostasis but there are very few data on the role of these cells in AD. Here, we investigated the presence of these cells in the blood of subjects with aMCI and mild AD (mAD) in comparison with healthy age-matched controls. We also quantitated several pro- and anti-inflammatory cytokines in sera which can influence the development and activation of these cells. We found significantly higher levels of MDSCs and Tregs in aMCI but not in mAD patients, as well as higher serum IL-1β levels. Stratifying the subjects based on CMV serostatus that is known to influence multiple immune parameters showed an absence of differences between aMCI subjects compared to mAD patients and healthy controls. We suggest that the increase in MDSCs and Tregs number in aMCI subjects may have a beneficial role in modulating inflammatory processes. However, this protective mechanism may have failed in mAD patients, allowing progression of the disease. This working hypothesis obviously requires testing in future studies.

## Introduction

Alzheimer disease (AD) is the most frequent neurodegenerative disease for which aging is the most important risk factor (1, 2). The pathogenesis of AD remains unknown. The paradigmatic amyloid beta hypothesis (3) is being increasingly challenged (4). Although AD is a chronic inflammatory disease that mainly involves the brain in its clinical manifestations, it is also a systemic disease (5). The preclinical stages of AD may last for decades before overt manifestations of the first symptoms are recognized. For instance, this is the case of amnestic mild cognitive impairment (aMCI) that is now termed mild neurocognitive disorder according to The Diagnostic and Statistical Manual of Mental Disorders (DSM 5) (6, 7). Thus, the first clinical step is the condition of aMCI which progresses to full clinical manifestations of AD in a number but not all individuals. Noticeably, the progression to full-blown AD is also relatively slow, occurring over a number of years (8).

Several immune alterations have recently been reported in AD patients (9). It was shown that the number of naïve T cells was relatively decreased, whereas the number of effector memory T cells was increased (10). We have reported that cells of the innate immune system were differentially altered in patients with aMCI compared to mild AD (mAD) (11). These data suggested that there was an upregulated inflammatory activity associated with some type of innate cells such as NK cells and neutrophils in aMCI. We have previously postulated that in the case of aMCI subjects, these cells respond to some still unidentified challenge that may originate from chronic viral, bacterial, or fungal infections. The recent demonstration that beta amyloid peptides, the most important component of the characteristic amyloid plaques in AD, possess antimicrobial properties (12–14) is consistent with this notion. Overall, these observations have raised the level of interest in immune changes associated with the development and progression of AD (9).

The activity of the immune system needs to be tightly controlled to provide a fast and targeted response to challenges, followed by inhibition of the response during the resolution phase to prevent chronic inflammation and tissue damage. Several types of immune regulatory cells participate to this essential regulatory mechanism and these include myeloid-derived suppressor cells (MDSCs) (15–17) and regulatory T cells (Tregs) (18–21). These cells suppress the immune response to prevent chronic inflammation and autoimmune processes (22, 23) although, in some cases, the response may be diverted in favor of a pathological process such as cancer (23–27).

Myeloid-derived suppressor cells are the most important immune modulatory cells of the innate immune system (28). These are essentially immature myeloid cells which may be either neutrophilic (CD15^+^) or monocytic (CD14^+^) (29, 30) and they composed a very heterogeneous population of cells (15). In humans, MDSCs are defined by the phenotype CD33^+^HLA-DR^−^ and are lineage (CD3, CD19, CD56)-negative (31). MDSCs suppress innate and acquired immune responses in cancer (31) and are elevated in chronic inflammation and malignancies (25, 32). It is of note that MDSCs have been found to be increased in healthy elderly subjects (28). In aging as well as in the case of chronic inflammatory states such as cardiovascular disease, cognitive decline, and frailty, pro-inflammatory mediators (TNF, IL-6, and IL-1β) production is commonly increased and is related to the differentiation of suppressor cells (33, 34). The role of MDSCs in normal physiology is complex. It has been reported that these cells impair the functions of T cells, NK cells, and dendritic cells through several pathways that include expression of arginase I, inducible nitric oxide synthase and Gp91phox and, the release of reactive oxygen species and peroxynitrite during antitumor immunity (26, 35, 36). MDSCs mainly suppress T cell function and NK cell cytotoxicity and they may also modulate macrophage polarization and, chemotaxis and functions of neutrophils (15, 16). MDSCs have been implicated not only in cancer but also in psoriasis (36), inflammatory bowel disease, traumatic stress, rheumatoid arthritis, and infections (37–44). It has also been shown that MDSCs may induce Tregs possessing a CD4^+^CD25^+^FoxP3^+^ phenotype in cancer settings such as hepatocellular carcinoma (45, 46).

Regulatory T cells (defined phenotypically in humans as CD4^+^FoxP3^+^) are essential to control immunity and self-tolerance. Accordingly, their dysregulation leads to unbalanced immune responsiveness, tissue damage and autoimmunity (23). Tregs are recognized as suppressors of host immune responses in antiviral immunity and promoters of tumor growth (18). Similarly to MDSCs, they consist of very heterogeneous cell populations in their origin and expression of different cell surface markers (47, 48). Their suppressive activity involves either or both cell-cell contact-dependent and cytokine-dependent (especially through secretion of IL-10 and TGFβ) action (21). Furthermore, they play a role in homeostasis and damage repair in non-lymphoid organs (47).

In addition to their essential role in lymphocyte homeostasis, Tregs play either detrimental or favorable roles in certain viral infections. Whereas Tregs have a detrimental role in chronic hepatitis C virus infection by contributing to viral pathogenicity (49), their role in HIV infection is equivocal, depending mostly on the clinical stage (50). Similarly, accumulation of Tregs is correlated with poor prognosis in many types of cancer including breast cancer and hepatocellular carcinoma (51). Tregs participation may be beneficial for reducing tumor progression and improving prognosis by suppressing the inflammatory activities of Th17 cells in colorectal cancers (52).

There are few reports on the role of Tregs in neurodegeneration and AD (53–55). In this connection, two studies in humans have reported an increase of Tregs in AD (54, 55). One of these studies reported that Tregs of aMCI patients were increased relative to AD patients (54). The authors concluded that the inflammatory process plays a major role in AD pathogenesis and that alterations of Tregs in AD patients may contribute to this pathology. However, the fundamental role of Tregs in AD has not been yet defined and it is not known whether they are beneficial by suppressing a specific immune-inflammatory response or whether they are harmful by suppressing a potentially beneficial immune response (56).

Accordingly, the role of MDSCs or Tregs in immune modulation may vary considering their development, phenotype, functions, and the pathological conditions under which they are activated (57). In the case of AD, it can be suggested that their activity may contribute to development and progression of a neuroinflammatory process by suppressing protective innate and adaptive immune functions mainly at the early stage of the disease. The fact that chronic inflammation is a progressive process in AD led us to investigate whether there were changes in the number of MDSCs and Tregs in aMCI or mAD patients. Modifications of the involvement of MDSCs and Tregs in AD may contribute to the suppression/modulation of the innate and adaptive immune responses. To answer this question, we quantitated the number of MDSCs and Tregs in the periphery of aMCI and mAD patients and compared the results to age-matched healthy elderly subjects. In addition, we quantitated the levels of various circulating cytokines, as potential drivers of the development of these cells. Results showed differential increases in the number of neutrophilic MDSCs and Tregs in aMCI subjects in contrast to healthy controls and mAD patients. IL-1β was the only pro-inflammatory cytokine that was increased in aMCI whereas IL-10 was decreased in both aMCI and mAD patients. Our data suggest that the increase in MDSCs and Tregs number in aMCI subjects may have a beneficial role in modulating inflammatory processes. However, this protective mechanism may have failed in mAD patients, allowing progression of the disease.

## Materials and Methods

### Subjects

Amnestic mild cognitive impairment and mAD diagnosis was made according to NINCDS-ADRDA criteria (57, 58) and the guidelines of Grundman et al. (59). Healthy elderly individuals satisfied the SENIEUR standard protocol for immuno-gerontological studies (60). Subjects who had a history or physical signs of atherosclerosis or inflammation were excluded. Evaluation and classification of subjects followed the standard protocol of our memory clinic including clinical, neuropsychological, and imaging assessment. Mini-mental state evaluation and Montreal cognitive assessment were performed, as we have previously described in details (11). The study included 11 healthy controls, 11 aMCI subjects, and 15 mAD patients. All subjects gave written informed consent in accordance with the Declaration of Helsinki. The protocol was approved by the Ethics Committee of the Faculty of Medicine and Health Sciences of the University of Sherbrooke (protocol # 2010-21/Fülöp). *Cytomegalovirus* (CMV) seropositivity was determined at the clinical laboratories of the Centre Hospitalier Universitaire de l’Université de Sherbrooke (CHUS) hospital. Additional details of the patients’ clinical data are summarized in Table 1.

**Table 1:** Patients’ clinical data.

Parameters		Healthy subjects (C) (*n* = 13)		Amnestic mild cognitive impairment (aMCI) patients (*n* = 13)		Mild AD (mAD) patients (*n* = 15)		*p* Values (Tukey’s posttest)		
				
Type	Units	Mean	SD	Mean	SD	Mean	SD	aMCI	mAD	aMCI
								versus C	versus C	versus mAD
Age	Years	71.1	5.22	72.8	3.64	78.1	4.37	0.50	<0.001	<0.01
Sex	Women (%)	77	–	70	–	80	–	–	–	–
	Men (%)	23	–	30	–	20	–	–	–	–
MMSE	Score/30	29.31	0.75	26.75	1.50	25.00	2.00	<0.05	<0.0001	<0.05
MoCA	Score/30	27.54	2.19	24.00	2.95	17.69	2.59	0.08	<0.0001	<0.0001
ApoE4	Frequency	0.35	–	0.67	–	0.39	–	–	–	–
WBC	10^9^/L	5.63	0.81	5.72	1.22	6.75	1.80	0.99	0.20	0.27
Lymphocytes (ab)	10^9^/L	1.72	0.36	1.62	0.38	1.90	0.66	0.92	0.72	0.48
Monocytes (ab)	10^9^/L	0.38	0.08	0.47	0.13	0.60	0.16	0.36	<0.01	0.11
PMN (ab)	10^9^/L	3.37	0.72	3.33	0.85	3.96	1.08	0.99	0.36	0.32
NLR	–	2.12	0.77	2.29	0.68	2.22	0.53	0.87	0.94	0.98
Hemoglobin	g/L	133.8	7.53	135.4	8.49	133.5	9.43	0.93	0.99	0.89
Total cholesterol	mmol/L	4.53	0.96	5.21	0.95	4.44	0.74	0.35	0.98	0.26
Triglycerides	mmol/L	1.72	0.79	1.72	0.79	1.71	0.50	0.99	0.99	0.99
HDL	mmol/L	1.49	0.39	1.69	0.40	1.48	0.49	0.68	0.99	0.64
LDL	mmol/L	2.41	0.56	3.01	0.66	2.27	0.60	0.20	0.90	0.10
TC/HDL	Ratio	3.13	0.58	3.22	0.70	3.50	1.18	0.98	0.68	0.83
CMV	Frequency	0.38	–	0.54	–	0.53	–	–	–	–
CRP	mg/L	1.20	1.85	0.79	1.45	0.99	1.55	0.90	0.97	0.98

*Serological parameters are from the clinical laboratories of the Centre Hospitalier Universitaire de l’Université de Sherbrooke (CHUS) and are analyzed by one-way analysis of variance and Tukey’s post-test. Absolute number is defined as the percentage of cells counted multiplied by the total number of white blood cells*.

*ab, absolute number; ApoE, apolipoprotein E; C, healthy (control) subjects; CMV *Cytomegalovirus* seropositivity; CRP, C-reactive protein; TC, total cholesterol; HDL, high-density lipoprotein; LDL, low-density lipoprotein; MMSE, mini-mental state evaluation; MoCA, Montreal cognitive assessment; NLR, neutrophil leukocyte ratio; PMN, polymorphonuclear neutrophils; WBC, white blood cells*.

### Cell Purification and Culture

Human peripheral blood mononuclear cells (PBMCs) were obtained from heparinized blood (80 ml) by density gradient centrifugation over Ficoll-Paque plus medium (GE Healthcare Life Sciences, Baie d’Urfé, QC) (10, 61). PBMCs were washed three times in phosphate-buffered saline (PBS) (Wisent, St. Bruno, QC) and resuspended in culture medium consisting of RPMI 1640, 10% fetal bovine serum and penicillin G (2.5 IU/ml) and streptomycin (50 µg/ml) (Wisent). PBMC viability was assessed by FACS analysis using near-IR LIVE/DEAD fixable kit (Life Technologies, Burlington, ON, Canada).

### Tregs and MDSC Analysis by FACS

Peripheral blood mononuclear cells (1 × 10^6^ cells) were washed twice with PBS (500 µl), suspended in PBS (1 ml) and stained with a LIVE/DEAD fixable Far Red IR Dead cells kit (1 µl) (Life Technologies Thermo Fisher Scientific, Waltham, MA) for 25 min at room temperature, in the dark for viability staining (data not shown). After washing twice (PBS 200 µl), cells were fixed using a fixation buffer (BioLegend, San Diego, CA, USA) containing 1% (w/v) paraformaldehyde (PFA) for 10 min at 4°C. Cells were treated for 10 min at 4°C with PBS containing 10% FBS to reduce non-specific binding. After washing twice with PBS, cells were incubated with the relevant antibody mix for 30 min at 4°C, in the dark. The antibody mix contained: CD3 brilliant violet 510 (BV510) (BD Biosciences), CD4 brilliant violet 421 (BV421) (BD Biosciences, San Jose, CA, USA), CD8 peridinin chlorophyll protein (PerCP) (BioLegend), CD25 allophycocyanin 7 (APC-Cy7) (BD Biosciences), CD28 alexa 700 (A700) (BioLegend), and CCR4 phycoerythrin-cyanin 7 (Pe-Cy7) (BD Bioscience). Permeabilization was performed according to the supplier’s instructions (eBioscience, Thermo Fisher Scientific) for Foxp3 and transcription factor permeabilization buffer kit, starting with incubation for 30 min in the dark with 200 µl of fixation and permeabilization buffer. After one wash with the permeabilization buffer (200 µl), intracellular staining with Foxp3 phycoerythrin (PE) (eBioscience) was performed for 30 min in permeabilization buffer. The last step of the staining procedure consisted in two washings with 200 µl of permeabilization buffer.

Myeloid-derived suppressor cell staining was performed on total blood freed of erythrocytes by hypotonic lysis (NH_4_Cl). Samples were fixed and saturated as described in the section above, then a staining step of 30 min with the antibody mix was done. The antibody mix contained the following antibodies: lineage allophycocyanin (APC) (BioLegend), CD33 fluorescein isothiocyanate (FITC) (BD Biosciences), CD11b phycoerythrin-CF594 (Pe-CF594) (BD Biosciences), HLA-DR brilliant violet 786 (BV786) (BD Biosciences), CD15 brilliant violet (BV510) (BD Biosciences). Then, two washings with 200 µl of PBS were performed. Cells were suspended in PBS, filtered through a nylon filter cloth (70 µm mesh size, Morgans Screening & Filters Ltd., Pickering, ON, Canada) to remove cell clumps and processed for analysis. Data were acquired on a FACSAria III (BD Biosciences) instrument using the FACSDiva v. 6.1 software. Analysis was performed using the FlowJo version 7.6.1 software (TreeStar, Ashland, OR, USA). Mean fluorescence intensity refers to the geometric mean of fluorescence intensity. An APC anti-human lineage cocktail (Lin 1) consisting of fluorescence-labeled monoclonal antibodies directed globally against CD3, CD19, CD20, and CD56 (BioLegend, product # 363601) was used to exclude these cells from MDSCs analysis. Examples of the strategy for cytofluorometric analysis are shown in Figure S1 in Supplementary Material.

### Cytokine Quantification in Sera

Sera were collected following centrifugation of heparinized blood (1,300 × *g*, 10 min). Samples were frozen at -80°C until the day of analysis using the Luminex technology (62). Levels of TNFα, IL-6, IL-1β, IL-10, IP-10, and IFNα in sera were quantified using a human cytokine magnetic bead assay (Milliplex^®^ MAP Multiplex Assays; EMD Millipore, Billerica, MA, USA). Quantification was performed according to the manufacturer’s instructions with a sample incubation step overnight, at 4°C. Data were acquired on a Luminex^®^ 200TM System using the Luminex xPonent^®^ software and analyzed using the Milliplex^®^ Analyst 5.1 software (EMD Millipore).

### Statistical Analysis

One-way analysis of variance (ANOVA) was used to test for differences among the three experimental groups. The single-step multiple comparison Tukey’s test was used in conjunction with ANOVA to assess differences in the means between the three experimental groups. Data were processed using the GraphPad Prism v 6.02 software (GraphPad Software, La Jolla, CA, USA).

## Results

### Myeloid-Derived Suppressor Cells

**Figure 1:**
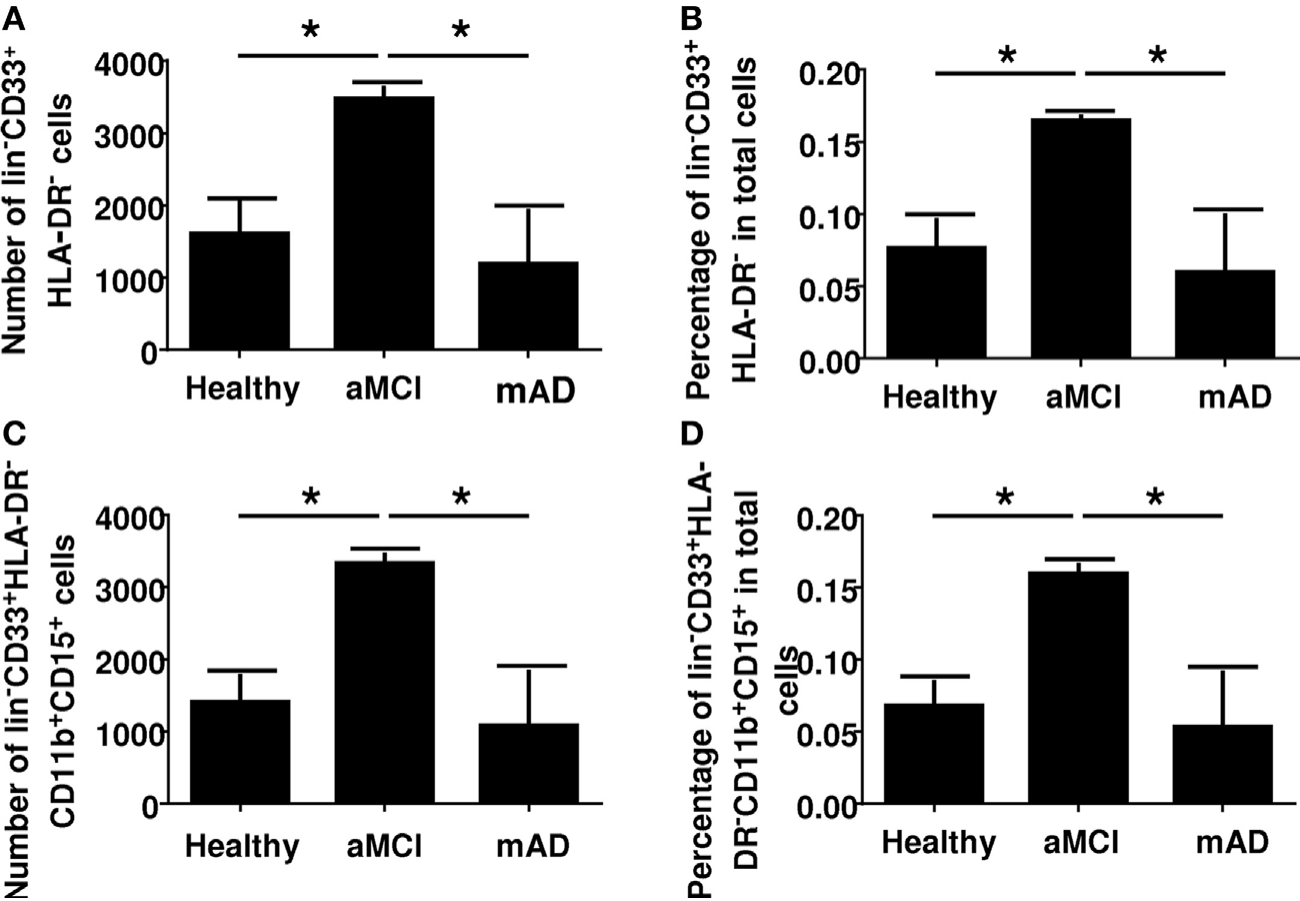
Determination of myeloid-derived suppressor cells number and percentage in whole blood of healthy, amnestic mild cognitive impairment (aMCI), and mild AD (mAD) subjects. **(A)** Number of CD33^+^HLA-DR^−^ cells in whole blood. **(B)** Percentage of CD33^+^HLA-DR^−^ cells in whole blood. **(C)** Number of CD33^+^HLA-DR^−^CD11b^+^CD15^+^ cells in whole blood. **(D)** Percentage of CD33^+^HLA-DR^−^CD11b^+^CD15^+^ cells in whole blood. Each group was composed of five independent subjects with determinations made in triplicate. Data are shown as the mean ± SEM. The asterisks (*) correspond to *p* < 0.05.

Myeloid-derived suppressor cells are immune regulatory cells of the myeloid lineage. They express CD33 but do not express HLA-DR. They are further characterized as originating from the neutrophil lineage by the expression of CD11 and CD15. We had found a complex differential change in innate cell functions (NK cells and neutrophils) in earlier studies. As a follow-up, we were interested to determine the phenotype of the common MDSC lineages and, more specifically, those derived from neutrophils since there is no data in the literature concerning the number of these cells in aMCI or mAD. Results showed a significant increase of MDSCs in the peripheral blood of aMCI subjects in contrast to healthy controls and mAD patients (Figure 1). For example, the number of CD33^+^HLA-DR^−^ cells in whole blood was significantly (*p* < 0.05) higher in aMCI subjects than mAD and control individuals (Figure 1A). In addition, the percentage of CD33^+^HLA-DR^−^ cells in whole blood was significantly (*p* < 0.05) higher in the aMCI group than the mAD and control groups (Figure 1B). Similar observations were made in the number (Figure 1C) and percentage (Figure 1D) of CD33^+^HLA-DR^−^CD11b^+^CD15^+^ cells in whole blood. Furthermore, these cells were nearly all of the neutrophil lineage. These observations clearly showed that aMCI subjects are characterized by increased levels of MDSCs, suggesting that an inflammatory condition may be downregulated in these individuals.

### Regulatory T Cells

**Figure 2:**
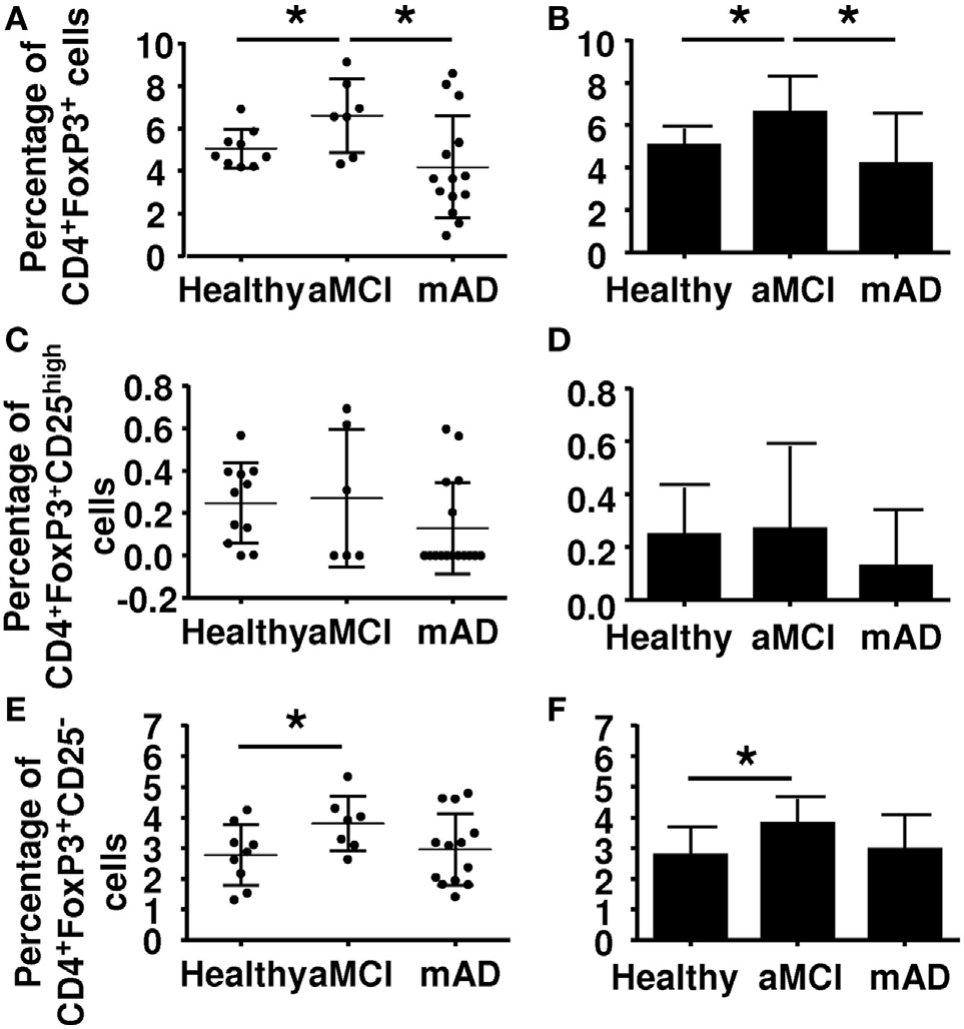
Determination of the percentage of regulatory T cells (Tregs) in peripheral blood mononuclear cell of healthy, amnestic mild cognitive impairment (aMCI), and mild AD (mAD) subjects. **(A)** Tregs percentages defined by the CD4^+^FoxP3^+^ markers in individual participants. **(B)** Corresponding histograms. **(C)** Tregs percentages defined by the CD4^+^Foxp3^+^CD25^high^ markers in individual participant. **(D)** Corresponding histograms. **(E)** T regs percentages defined by CD4^+^Foxp3^+^CD25^−^ markers in individual participants. **(F)** Corresponding histograms. Each group was composed of at least five independent subjects with determinations made in triplicate. Data are shown as the mean ± SEM. The asterisks (*) correspond to *p* < 0.05.

Regulatory T cells are immunomodulatory cell derived from the CD4^+^ Th1 lineage. They originate from thymus-derived CD4^+^ T cells. They are characterized by expression of FoxP3 and CD25. Classically, Tregs express high levels of CD25, although the possibility cannot be excluded that this characteristic reflects a state of activation. Whereas Tregs suppress the hyperreactivity of the immune system by inhibiting autoimmune reactions, they also interfere with dysregulation of the effective immune response in aging. Here, results showed that the number of FoxP3^+^ CD4^+^ T cells was significantly increased in aMCI subjects compared to healthy controls and mAD patients (Figures 2A,B). However, double labeling using the additional CD25 marker showed an absence of differences between the three experimental groups (Figures 2C,D), suggesting that CD25 was only a marker of activation in these cells. In this connection, a newly discovered Treg subtype which is Foxp3^+^ but CD25^−^ has been reported to be increased in elderly subjects (48) and found here to be increased in aMCI subjects (Figures 2E,F).

**Figure 3:**
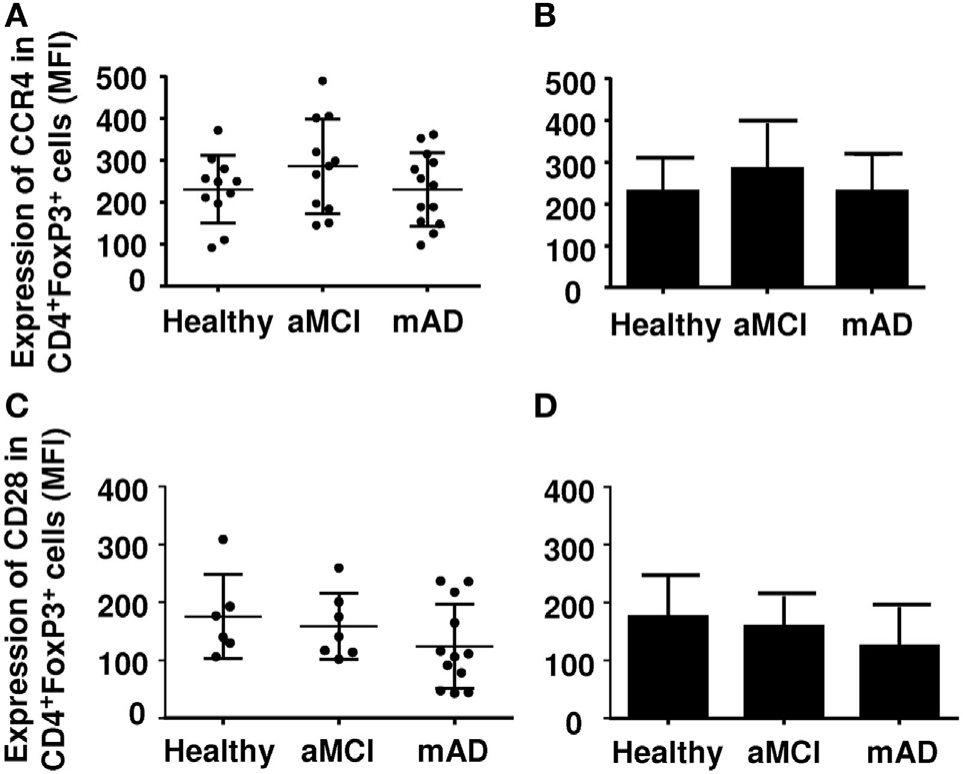
Expression of CCR4 and CD28 on regulatory T cells (Tregs) of healthy, amnestic mild cognitive impairment (aMCI), and mild AD (mAD) subjects. **(A)** CCR4 number in Tregs defined by the CD4^+^FoxP3^+^ marker in individual participants. **(B)** Corresponding histograms. **(C)** CD28 number in Tregs defined by the CD4^+^Foxp3^+^CD25^high^ marker in individual participants. **(D)** Corresponding histograms. Each group was composed of at least five independent subjects with determinations made in triplicate. Data are shown as the mean ± SEM.

**Figure 4:**
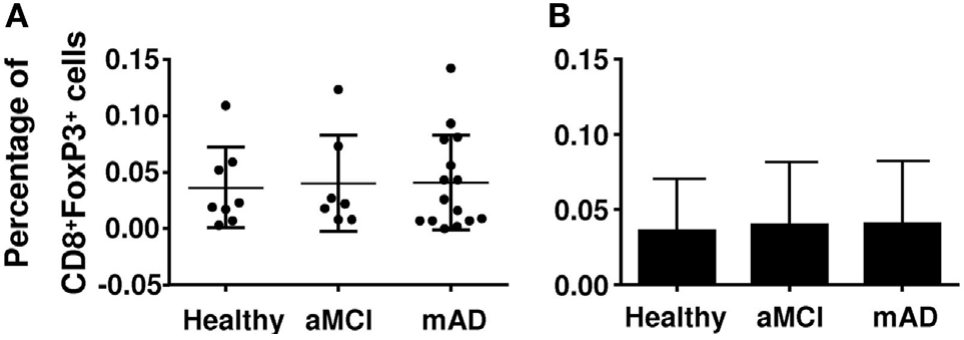
Quantification of the percentage of CD8^+^ regulatory T cells (Tregs) in healthy, amnestic mild cognitive impairment (aMCI), and mild AD (mAD) subjects. **(A)** Percentage of CD8^+^FoxP3^+^ Tregs in individual participants. **(B)** Corresponding histograms. Each group was composed of at least five independent subjects with determinations made in triplicate. Data are shown as the mean ± SEM.

Regulatory T cells also express other markers which may be useful in distinguishing their nature and activity. The chemokine receptor CCR4 has been reported be increased in mAD patients, potentially facilitating homing of these cells in the brain. Here, CCR4 (Figures 3A,B) and CD28 (Figures 3C,D) expression was similar in the three experimental groups. Analysis of potentially suppressive FoxP3^+^CD8^+^ T cells showed that expression did not differ between the three experimental groups (Figures 4A,B).

### Circulating Cytokine Levels

**Figure 5:**
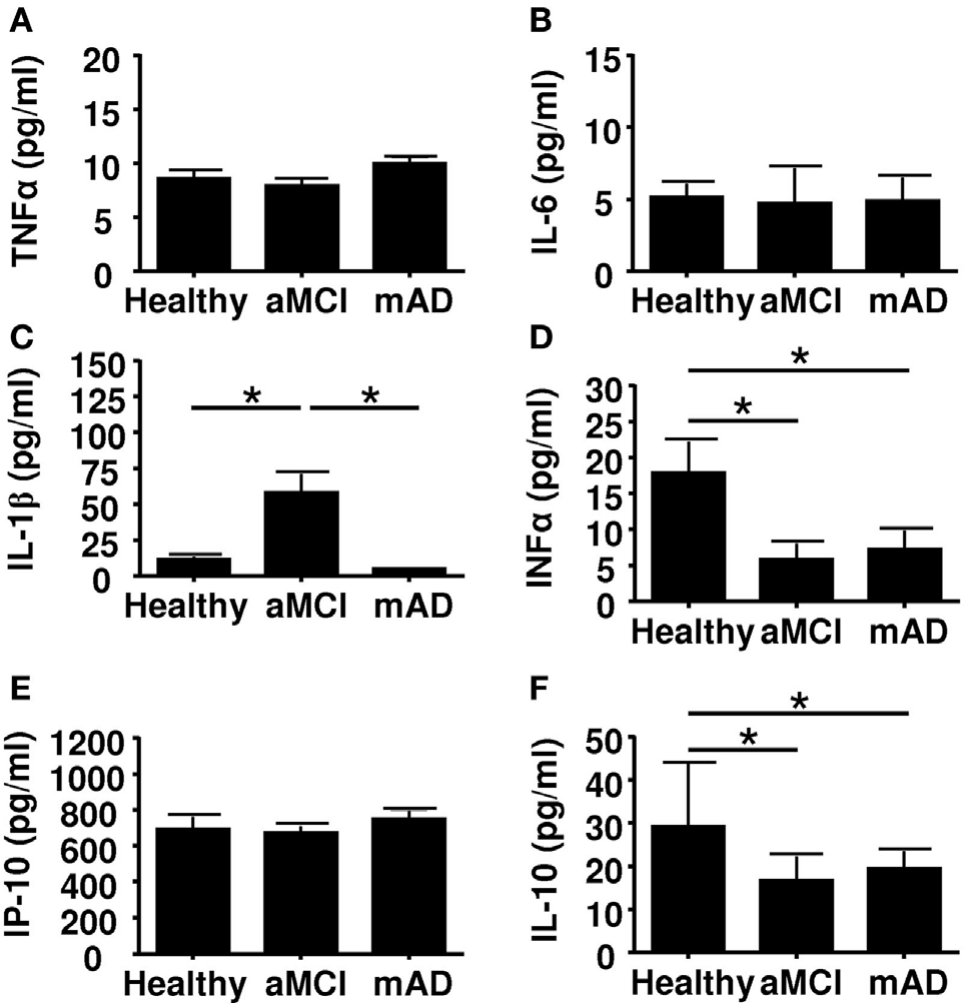
Quantification of cytokine concentrations in sera of healthy, amnestic mild cognitive impairment (aMCI), and mild AD (mAD) subjects. **(A)** TNFα. **(B)** IL-6. **(C)** IL-1β. **(D)** Il-10. **(E)** IP-10. **(F)** IFNα. Each group was composed of at least 10 independent subjects with determinations made in triplicate. Cytokine quantification was done using the Luminex technology. Data are shown as the mean ± SEM. The asterisks (*) correspond to *p* < 0.01.

Alzheimer disease may be viewed as a chronic inflammatory state with increased pro-inflammatory cytokines in circulation (63). To test the possibility that these cytokines influenced immunomodulatory activity of MDSCs and Tregs, we quantified the circulating levels of the pro-inflammatory cytokines TNFα, IL-6, IL-1β, IFNα, and IP-10. Results showed that the serum levels of TNFα and IL-6 were similar in the three experimental groups (Figures 5A,B). In marked contrast, the circulating levels of IL-1β were significantly higher (*p* < 0.01) in aMCI subjects than healthy controls and mAD patients (Figure 5C). With respect to the levels of the anti-inflammatory cytokine IL-10, data showed that these were higher in healthy subjects than in aMCI and mAD patients (Figure 5D). Whereas the concentration of IP-10 did not significantly differed between the three experimental groups (Figure 5E), it was significantly (*p* < 0.01) higher in healthy controls than in aMCI and mAD patients (Figure 5F). The bulk of these observations suggested that IL-1β, an important pro-inflammatory cytokine resulting from the inflammasome stimulation *via* pathogen recognition receptor stimulation, is specifically increased in aMCI. Furthermore, this observation suggested that IL-1β may play a beneficial role in aMCI but its increased production could also detrimental as it has been reported at the AD stage. In this connection, it has been shown that IL-1β specifically impairs microglial clearance of Aβ in AD (64, 65).

**Figure 6:**
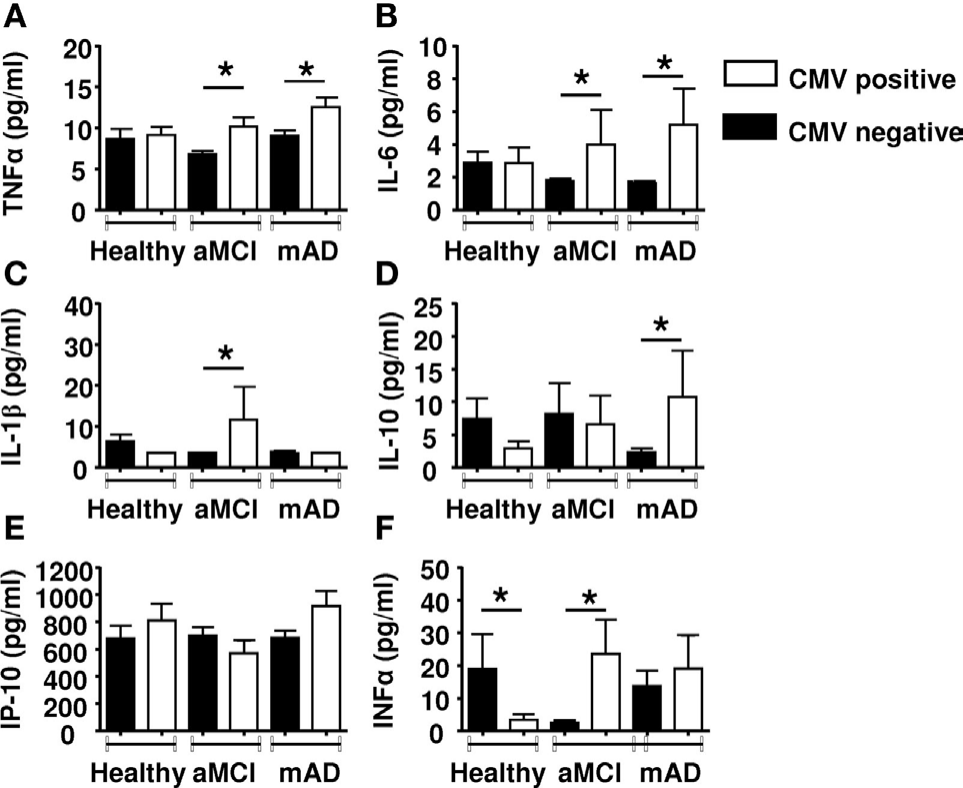
Quantification of cytokine concentrations in sera according to CMV serostatus. **(A)** TNFα. **(B)** IL-6. **(C)** IL-1β. **(D)** Il-10. **(E)** IP-10. **(F)** IFNα. Each group (healthy controls, amnestic mild cognitive impairment [aMCI], and mild AD [mAD]) was composed of at least 10 independent subjects with determinations made in triplicate. Cytokine quantification was done using the Luminex technology. Data are shown as the mean ± SEM. The asterisks (*) corresponds to *p* < 0.01. Empty rectangle, CMV-positive cells, filled rectangle, CMV-negative cells.

We stratified the subjects according to CMV-seropostivity. It has been reported that latent infection with this herpesvirus influences several peripheral immune parameters (66). There was nearly the same number of subjects CMV^+^ or CMV^−^ in each group. Data revealed significant (*p* < 0.01) increases in the levels of pro-inflammatory cytokines TNFα (Figure 6A) and IL-6 (Figure 6B) in CMV-positive aMCI and mAD subjects. However, levels of pro-inflammatory cytokine IL-1β were significantly higher (*p* < 0.01) only in the case of CMV-positive aMCI subjects (Figure 6C). In contrast, the serum concentration of IL-10 was significantly (*p* < 0.01) elevated only in the case of CMV-positive mAD patients (Figure 6D). The levels of IP-10 were not influenced by CMV serostatus (Figure 6E). Whereas the levels of IFNα were lower in CMV-positive healthy subjects, they were significantly (*p* < 0.01) higher in CMV-positive aMCI subjects but similar in mAD patients (Figure 6F). Overall, the bulk of the results suggested that CMV seropositivity influenced the production of pro- and anti-inflammatory cytokines in aMCI and mAD patients.

## Discussion

Although the cause(s) of the AD remains controversial, several immune-related alterations have been documented (9–11). The contribution of neuroinflammation to the pathogenesis of the disease has been acknowledged for some time, although the trigger(s) that sustains a state of inflammation is still uncertain (63). Recent observations have put forward convincing evidence that amyloid beta peptides possess antimicrobial activity (12–14, 67–69), suggesting that AD may have an infectious origin (69). In addition, there are data regarding the role of effector cell functions in AD (11, 70) but, to the best of our knowledge, the frequency of circulating MDSCs has not been reported. Here, we found an increase in total MDSCs, especially in the neutrophilic subset (PMN-MDSCs) in the aMCI group but not in the mAD and healthy groups (Figure 1). It is of note that the number of monocytic MDSCs were similar in the three experimental groups (data not shown). In these cases, percentages of monocytic MDSCs in whole blood or in PBMCs were similar between the three experimental groups, as reported by others in whole blood or PBMCs (16). It is of note that an increase in the number of MDSCs has been reported in peripheral blood of healthy aged and frail elderly patients (28). In many cancer patients, similar increases in MDSCs have been observed and their number correlated with the clinical outcomes as well as response to immunotherapy (68, 69, 71, 72). Similar increases have been reported in various bacterial and viral infections, mainly in the maintenance of chronicity (31). In these settings, the increased differentiation of these cells was correlated with increased levels of circulating pro-inflammatory cytokines (39). Here, we found only an increase in the levels of IL-1β in aMCI patients which may partly explain these observations, since IL-1 may mediate its effects by stimulating NO production as a result of increased iNOS expression (73). Our findings correlate with those that showed that IL-1β was increased in the AD brain as well as after Aβ stimulation and has a clinical stage dependent effect however mainly described as mediating neurotoxicity (74, 75). Furthermore, Il-1β has been shown to induce MDCSs accumulation and differentiation (16, 33, 76). It is also of interest, as infection was raised as a possible cause of AD, immunocompetent individuals rarely develop pro-inflammatory antifungal immune responses because as among many other pro-inflammatory IL1-β mediates MDSCs recruitment and modulate antifungal immune response (77). It is of note that no other pro-inflammatory cytokines were found to be increased in any of the experimental groups (Figure 5). The anti-inflammatory cytokine effect could not be established here, but may not be sufficient to compensate for the increase of IL-1β. We investigated the influence of CMV serostatus on cytokine production. We observed an upregulated production of pro-inflammatory cytokines (TNFα, IL-6, and IL-1β) in the aMCI and mAD groups. These observations suggested that CMV serostatus did not allow a distinction regarding production of pro-inflammatory cytokines between the two clinical stages of the disease studied here.

We observed an increase in Tregs in the aMCI group compared to healthy and mAD subjects (Figure 2). These observations are in agreement with the report of higher frequency of Tregs in elderly individuals and suppressive activity in neurodegeneration (55). However, when analysis of expression of the CD25 surface marker was done as a marker of CD4^+^FoxP3^+^CD25^high^ in the antibody staining panel, results showed the absence of significant differences among the three experimental groups. This observation suggested that CD25 is an activation marker which cannot further distinguish between Tregs subpopulations. Interestingly, the newly characterized CD25-negative Treg subpopulation has been reported to be increased in aMCI subjects (48, 78). Of interest, this Treg subpopulation has also been found to be increased in SLE and in aging mice and shown to correlate with decreased T cell responses (78). Here, the expression of the surface markers CD28, CCR4 was found to be similar between the three groups of subjects. Together, the differences reported between aMCI and mAD patients concerning Tregs confirmed previous data with respect to the increased Treg specifically in aMCI (54). In this report, the authors further showed that in aMCI subjects, the increase in Tregs numbers was associated with upregulated suppressive activity toward T cell functions, which was decreased in AD patients. In the present study, we could not investigate the functions of Tregs because of an insufficient number of cells from each donor. This hurdle prevented us investigate, at this time, the influence of Tregs increase in aMCI subjects. However, it is to be noted that the increase of MDSCs was paralleled by the increase of Tregs as well.

There is no clear functional explanation for the increased number of MDSCs in aMCI subjects and the decrease in mAD patients. However, in the case of autoimmune diseases, their number has been reported to be increased, presumably as a mean to compensate for uncontrolled immune responses (36). This behavior can be a self-regulatory mechanism whereby a chronic inflammatory response induces the increase of immunoregulatory cells in an attempt to downregulate the hyper-response, even if an effector immunoparalysis appears concomitantly (11, 79). In cases of aMCI where there is still a strong inflammatory response, as demonstrated for NK cells and neutrophils, the increase in MDSCs may appear to be a compensatory mechanism. However, this situation may create a vicious cycle resulting in the increase of inflammation, along with progression of the disease toward AD where the immune response is already less inflammatory (9, 11). This possibility has been put forward in the case of Tregs by Saresella et al. (54). These authors have suggested that the increase in Tregs in aMCI subjects occurs as a mean to decrease uncontrolled inflammation, whereas the decrease in AD favors uncontrolled progression of the disease. Our recent studies on the immune response in aging and in various stress situations of elderly also support this hypothesis (79). However, an alternative explanation cannot be excluded, namely, that inflammation in aMCI subjects is still a necessary process to retard the deleterious effect of progression toward AD as was proposed in a murine model of AD (53). If this were the case, the involvement of MDSCs and Tregs may be harmful. More investigations are obviously needed to explore the mutual balanced effects of MDSCs and Tregs on the intrinsic and extrinsic disease environment. Until this question is resolved, all tentative efforts to modulate these cells in aMCI may be counterproductive or even dangerous.

## Author Contributions

TF participated in the recruitment of subjects, the conceptualization, the interpretation of the data, and writing of the paper. ALP has realized the experiments, analyzed the data and participated in the writing of the manuscript. HG has made the cytokine measurements by Luminex and participated in the analysis of the data. EF, AL, GD, JW, and GP have participated in the conceptualization, the interpretation of the data, and writing of the paper.

## Conflict of Interest Statement

The authors declare that the research was conducted in the absence of any commercial or financial relationships that could be construed as a potential conflict of interest.

## Supplementary Material

Figure S1 Gating strategy used for analysis of regulatory T cells (Tregs) and myeloid-derived suppressor cells (MDSCs) by cytofluorometry. Far Red is a dye used to stain live cells (see Section “Materials and Methods” in the main text). Lin refers to an APC anti-human lineage cocktail (Lin 1) consisting of fluorescence-labeled monoclonal antibodies directed globally against CD3, CD19, CD20 and CD56 (see Section “Materials and Methods” in the main text). Abbreviations are: FSC, forward side scatter; SSC, size side scatter.
