# Supplementary figures and images for: Differential Phenotypes of Myeloid-Derived Suppressor and T Regulatory Cells and Cytokine Levels in Amnestic Mild Cognitive Impairment Subjects Compared to Mild Alzheimer Diseased Patients
Source: Front Immunol. 2017 Jul 7;8:783. doi: 10.3389/fimmu.2017.00783 (PMC5500623; doi:10.3389/fimmu.2017.00783)

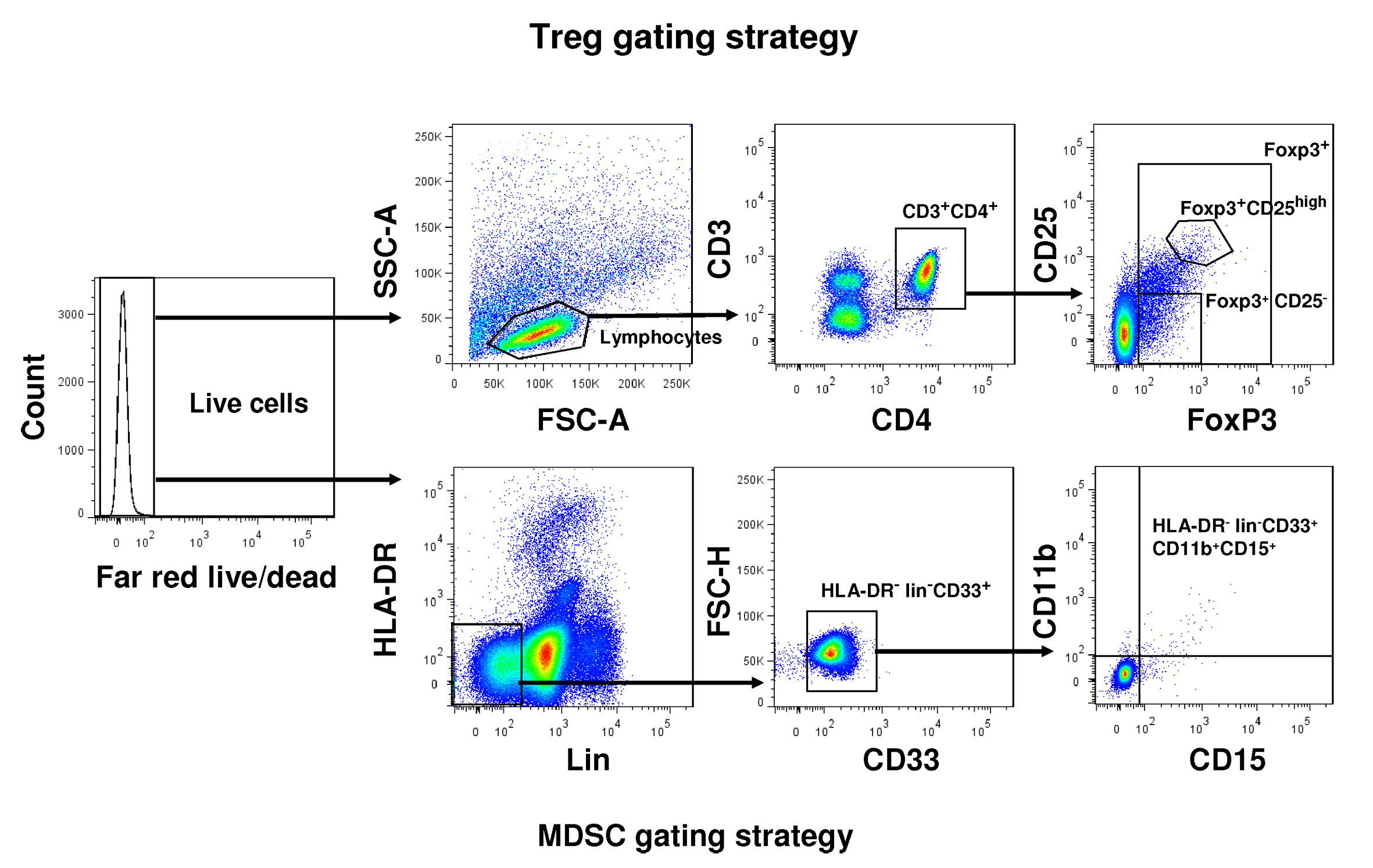

Supplement: Figure S1 — Gating strategy used for analysis of regulatory T cells (Tregs) and myeloid-derived suppressor cells (MDSCs) by cytofluorometry. Far Red is a dye used to stain live cells (see Section “Materials and Methods” in the main text). Lin refers to an APC anti-human lineage cocktail (Lin 1) consisting of fluorescence-labeled monoclonal antibodies directed globally against CD3, CD19, CD20 and CD56 (see Section “Materials and Methods” in the main text). Abbreviations are: FSC, forward side scatter; SSC, size side scatter. [file Image_1.TIF]
